# Supplementary figures and images for: miR-455-3p has superior diagnostic potential to PSA in peripheral blood for prostate cancer
Source: PLoS One. 2025 Feb 14;20(2):e0317385. doi: 10.1371/journal.pone.0317385 (PMC11828392; doi:10.1371/journal.pone.0317385)

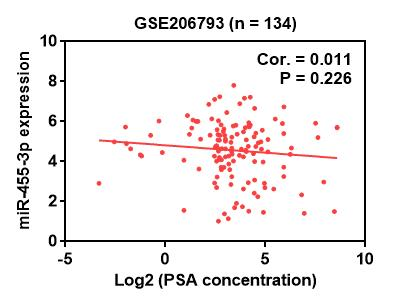

Supplement: S1 Fig — (TIF) [file pone.0317385.s001.tif]

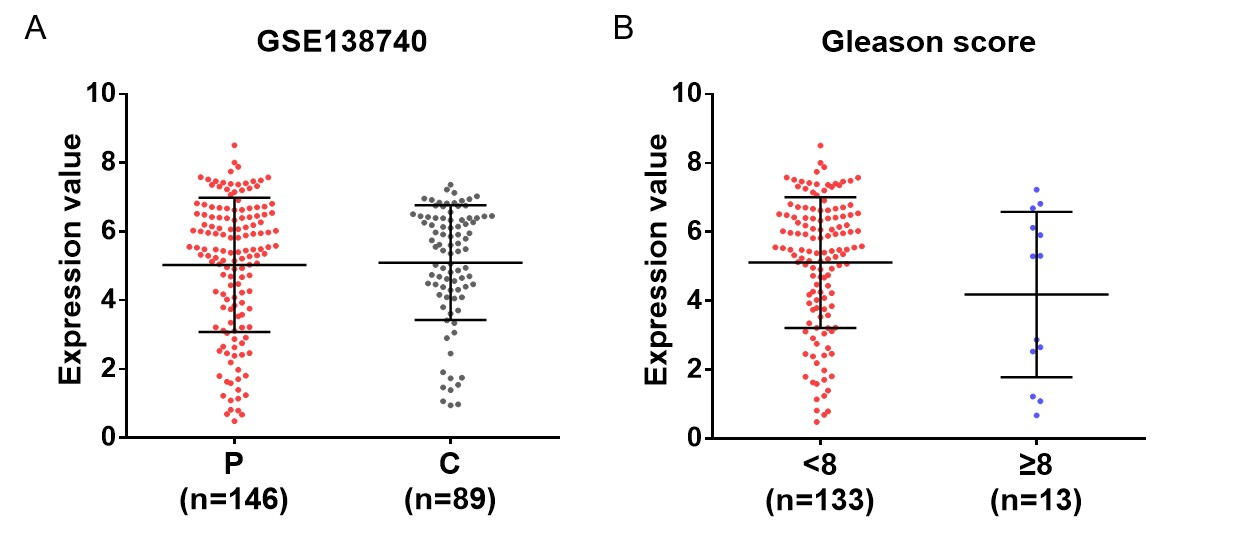

Supplement: S2 Fig — (A) Comparison of miR-455-3p expression between P and C groups in GSE138740 dataset. (B) Comparison of miR-455-3p expression between patients with GS < 8 and ≥ 8 in GSE138740 dataset. (TIF) [file pone.0317385.s002.tif]
