# Supplementary material for: miR-455-3p has superior diagnostic potential to PSA in peripheral blood for prostate cancer
Source: PLoS One. 2025 Feb 14;20(2):e0317385. doi: 10.1371/journal.pone.0317385 (PMC11828392; doi:10.1371/journal.pone.0317385)
Supplement: Attachment — Submitted filename: response.docx [file pone.0317385.s004.docx]

Please determine the internal control and housekeeping gene in the material method.

Thank you for your feedback. We have revised the manuscript accordingly. In the qPCR analysis, we have included U6 as the internal control gene to normalize the expression levels. This modification is now reflected in the materials and methods section.

Please use the reference for the Livak method (2-∆∆CT).

Thank you for your suggestion. We have now included the appropriate reference for the Livak method (2^-ΔΔCT) in the manuscript:

Schmittgen TD, Livak KJ. Analyzing real-time PCR data by the comparative C(T) method. Nat Protoc. 2008;3(6):1101-8. doi: 10.1038/nprot.2008.73. PMID: 18546601.

It would be better to check urine Cell-Free MicroRNAs in prostate cancer patients and compare it with serum concentration.

Thank you for the insightful suggestion. We have incorporated an analysis of urine cell-free miRNAs by adding GSE138740 dataset: Additionally, we included Figure S2, which presents correlation analyses of urinary cell-free miR-455-3p with clinical features in prostate cancer (PCa) patients. Specifically:

**Figure S2A** shows a comparison of miR-455-3p expression between prostate cancer (P) and control (C) groups in the GSE138740 dataset.

**Figure S2B** compares miR-455-3p expression between patients with Gleason scores (GS) < 8 and ≥ 8 in the GSE138740 dataset.

Please explain the number of patients and healthy control in the material method, in sample collection the number of patients is not enough (PCa:8, healthy:4); however, in the result it was much more, so clarify the number of samples in the Clinical Sample Collection. Also, mention if qPCR was conducted on all the samples.

Thank you for pointing this out. In the revised materials and methods section, we have clarified the number of samples used in our analysis:

The results presented in Figures 1–3 and 5 are based on analyses of public datasets (GSE206793 and GSE112264). Figure 4 results reflect analyses conducted on clinical samples, which included qPCR validation.

For the clinical sample collection, we used a total of 8 prostate cancer (PCa) samples and 4 healthy controls for qPCR analysis, as described. The qPCR was conducted on all clinical samples collected, and this has been clarified in the materials and methods section.
It would better to estimate the correlation (r) between the concentration of PSA and the relative gene expression value in patients and healthy control.

Thank you for the suggestion. We have conducted a correlation analysis between PSA concentration and miR-455-3p expression and presented the results in **Figure S1**, which shows the correlation in the GSE206793 dataset.
